# School Principals’ Perspectives and Leadership Styles for Digital Transformation: A Q-Methodology Study

**DOI:** 10.3390/bs16020165

**Published:** 2026-01-24

**Authors:** Peili Yuan, Xinshen Chen, Huan Song

**Affiliations:** Center for Teacher Education Research, Beijing Normal University, Beijing 100875, China; yuanpeili@mail.bnu.edu.cn (P.Y.); 202431010065@mail.bnu.edu.cn (X.C.)

**Keywords:** principal leadership, school digital transformation, Q methodology, GenAI, China

## Abstract

The advent of generative AI (GenAI) and its growing use in education has sparked a renewed wave of school digital transformation. School principals are pivotal in advancing and shaping school digital transformation, yet little is known about how they understand and lead digital transformation in the age of GenAI, particularly within China’s complex educational system. This study employed Q methodology to identify the perceptions and leadership styles of Chinese K–12 school principals toward school digital transformation in the age of GenAI. An analysis of a 30-item Q set with a P sample of 23 principals revealed four leadership types: Cautious Observation–Technological Gatekeeping Leadership, Moderate Ambition–Culturally Transformative Leadership, Moderate Ambition–Emotionally Empowering Leadership, and High Aspiration–Strategy-Driven Leadership. Overall, principals’ stances on GenAI formed a continuum, ranging from cautious observation and skeptical optimism to active embrace. These perceptions and leadership styles were shaped by Confucian cultural values, a flexible central–local governance arrangement, and parents’ high expectations for students’ academic achievement. Furthermore, structural constraints in resource provision further heightened principals’ reliance on maintaining guanxi-based relationships. This study enhances the understanding of the diversity of principals’ leadership practices worldwide and offers actionable insights for governments and principals to more effectively advance AI-enabled school digital transformation.

## 1. Introduction

Digital technologies have become a critical resource within education systems, offering significant opportunities to enhance various aspects of school development (Feroz et al., 2021). The process of profoundly transforming an organization’s activities, boundaries, and goals in order to leverage the opportunities brought by digital technologies is commonly referred to as digital transformation (Matt et al., 2015; Vial, 2019). The COVID-19 pandemic exposed the uneven digital preparedness of schools and accelerated the process of school digital transformation globally (Iivari et al., 2020; Boeskens & Meyer, 2025). More recently, generative artificial intelligence (GenAI) has moved from rapid public mainstream adoption to increasingly widespread permeation in school settings, with particularly rapid expansion since late 2022 (Jauhiainen & Guerra, 2023; Lim et al., 2025). This shift has increased the urgency of school digital transformation for most educational systems worldwide (Al Nuaimi et al., 2024; Boeskens & Meyer, 2025).

School principals are at the center of decision-making regarding the acceleration of school digital transformation (Reis-Andersson, 2024; Al Nuaimi et al., 2024). Thus, before integrating new innovative tools into schooling, it is crucial to understand the awareness and leadership styles of school leaders (Carr, 2011; Holmström, 2022; Pietsch & Mah, 2025). GenAI refers to technologies that learn patterns from large-scale data and generate novel multimodal content, such as text, images, and audio, in response to user prompts (Feuerriegel et al., 2024). Although AI is not new and has been applied in education for decades (Rismanchian & Doroudi, 2025), the integration of GenAI into educational practice is often marked by substantial uncertainty and opacity (Lim et al., 2025). This integration may intensify existing ethical concerns and generate new risks, including those related to accountability, fairness, privacy, and responsible use (OECD, 2023; Kaplan-Rakowski et al., 2023; Nordström, 2022). As AI-enabled schooling expands, established assumptions about teaching, learning, and school governance become less tenable, prompting leaders to recalibrate their practices and decision-making amid greater ambiguity (Azorín & Fullan, 2022; Fullan et al., 2024; Lim et al., 2025). Empirical research by the American Federation of School Administrators (2023) demonstrates that AI has reshaped principals’ work across multiple critical domains, including data analysis, administrative automation, and resource optimization. Accordingly, principals must navigate heightened uncertainty and make high-stakes decisions about adoption, governance, and risk mitigation in the face of unprecedented, and sometimes competing, demands (Nguyen et al., 2023; Singh et al., 2024).

As one of the world’s largest and most dynamic education systems (Yan, 2024), China offers a distinctive context for investigating principals’ leadership in school digital transformation in the age of GenAI (Li et al., 2025). The Chinese government has demonstrated strong commitment to advancing AI-driven educational transformation through a series of national policy documents, including China’s Education Modernization 2035 (State Council of the People’s Republic of China, 2019) and the Education Power Construction Master Plan (2024–2035) (Central Committee of the Communist Party of China & State Council of the People’s Republic of China, 2024). These policies emphasize the full implementation of a national digital education strategy and position the deep integration of digital technologies, particularly GenAI, as a key lever for improving educational quality. While the top-down policy vision is clear, how principals translate this vision into school-level practice remains complex and underexamined.

Over the past several decades, research conducted largely in developed-country settings has substantially advanced our understanding of how school leaders shape and influence school digital transformation (Sproule & Mombourquette, 2020; Banoğlu et al., 2023; Azorín & Fullan, 2022). Despite these advances, research on leadership for school transformation remains largely grounded in Euro-American educational contexts and crucially, much of it predates the rapid diffusion of GenAI in education (Shamir-Inbal & Blau, 2021; Karakose et al., 2021; Navaridas-Nalda et al., 2020). Moreover, leadership theories developed in Western contexts often presume relatively high levels of school autonomy and stable institutional boundaries, and therefore do not fully capture the realities of principal leadership in China (Li et al., 2025; Wang et al., 2025). Consequently, empirical evidence on Chinese principals’ leadership practices in school digital transformation in the age of GenAI remains comparatively scarce. This gap matters because Chinese principals are on the frontline of national reforms while navigating resource constraints, high parental expectations, and deeply rooted cultural traditions, pressures that may intensify in the age of GenAI (Li et al., 2025; Wang et al., 2025).

To address this gap, this study employs Q methodology (hereafter Q) to identify distinct patterns in Chinese K–12 principals’ perceptions of AI-driven school digital transformation and to determine their leadership styles. Q is designed to systematically capture subjectivity, including individuals’attitudes, perspectives, and beliefs, toward novel or complex phenomena through small sample sizes (Rodl et al., 2020; Lim-Ratnam et al., 2022). As Lundberg et al. (2020) note, Q methodology captures individuals’ holistic subjective configurations, addressing a key limitation of conventional quantitative approaches whereby an emphasis on averages can obscure minority or marginalized perspectives. Therefore, Q methodology is particularly well suited to identifying distinct perception–leadership types among principals, revealing how they make sense of school digital transformation in the age of GenAI and enact leadership practices in response.

This study addresses the following research questions:

RQ1. What distinct types of perceptions and corresponding leadership styles do Chinese K–12 principals exhibit regarding school digital transformation in the age of GenAI?
RQ2. What are the key characteristics associated with each type?

## 2. Literature Review

### 2.1. Reconceptualizing School Digital Transformation in the Age of GenAI

School digital transformation refers to the integration of digital technologies into school systems, reshaping educational practices, organizational structures, and the broader educational ecosystem (Qualter, 2024; Hinings et al., 2018). It encompasses how digital tools facilitate the collection, analysis, and utilization of educational data (Jarke & Breiter, 2019). Furthermore, it involves adapting teaching practices and institutional cultures to accommodate the integration of technology (Consoli et al., 2023; Timotheou et al., 2023). School digital transformation also entails ensuring equitable access to devices and reliable internet, safeguarding online safety, privacy, and data security, and advancing accessibility and inclusion for diverse learners, including rural students and those with disabilities or special educational needs (Chanenson et al., 2023; UNESCO, 2023). The goal of digital transformation is to enhance educational outcomes and efficiency, achieving equitable, sustainable, and learner-centered education, while helping students better navigate the challenges posed by broader societal digital transformation (Voogt et al., 2013; OECD, 2023; Timotheou et al., 2023).

In the wave of digital transformation, technological evolution remains the core driving force (Nhung & Huong, 2025). Educational technologies often follow major product cycles of roughly 36 months, whereas evaluation and accountability mechanisms lag behind, leaving assessment processes unable to keep pace with technological change (OECD, 2023; UNESCO, 2023). The past three decades have seen a tremendous increase in the technology available in schools (Bice et al., 2022). Early ICT-based digitization primarily involved the digitization of education-related materials, with technology mainly being used for information storage and retrieval (Nhung & Huong, 2025). Today, GenAI not only transforms digital learning resources from static repositories into dynamic adaptive educational tools (Chen et al., 2020) but also reshapes the very essence of teaching and learning through applications such as intelligent tutoring, personalized learning paths, and automated assessment (Lim et al., 2025; Giannakos et al., 2025). However, this transformation is accompanied by high levels of uncertainty, raising a series of pressing ethical, technical, and practical challenges (Holmes et al., 2019). These include issues such as academic integrity (Lee et al., 2024; Kofinas et al., 2025) and impacts on cognitive development (Zhao et al., 2024). Organizations may also face challenges when implementing GenAI. For instance, algorithmic opacity can hinder accountability (Langer & König, 2023), raise concerns about fairness among organizational members (Carter, 2020), and diminish decision-making performance (Yeomans et al., 2019). Furthermore, GenAI usage may undermine individual autonomy and motivation at work (Vaassen, 2022). Overall, GenAI is increasingly positioned as a transformative technology for education, yet empirical evidence for such claims remains limited. Meanwhile, its diffusion may introduce or amplify concerns beyond the classroom, including privacy risks and environmental externalities such as electronic waste and increased energy consumption (UNESCO, 2023). With all of these positive and negative possibilities, principal leadership requires consideration of the role of school leaders within this new educational landscape (Fullan et al., 2024; Pietsch & Mah, 2025).

### 2.2. Principal Leadership for School Digital Transformation

The success of digital transformation depends not only on technological infrastructure but also on the coordinated transformation of school policies, culture, and leadership roles (Pettersson, 2021; Reis-Andersson, 2024; Al Nuaimi et al., 2024; Ghavifekr & Pei, 2023). Over recent decades, scholarship on school leadership has increasingly moved away from rigid, directive approaches toward more adaptive and collaborative models (Li et al., 2025). Distributed leadership decentralizes responsibilities to promote shared decision-making and professional collaboration (Nadeem, 2024). In parallel, learning-centered leadership has emphasized continuous learning, evidence-informed instructional improvement, and the development of a school-wide culture for professional growth. More recently, the notion of digital leadership has been advanced to specify the leadership capacities and practices required to steer school change in technology-rich environments. Digital leadership extends rather than replaces traditional leadership models. It foregrounds principals’ digital competence and purposeful technology use, a lifelong-learning orientation, sustained technical support and professional development for teachers, and a coherent vision for school digital transformation (Ghavifekr & Pei, 2023; Karakose et al., 2021). This evolution provides an important conceptual foundation for understanding principal leadership (Suhaimi et al., 2025).

Among the many leadership models applied to digital transformation, transformational leadership has frequently been portrayed as particularly suitable for uncertain environments because it can articulate a shared vision, provide intellectual stimulation, and offer individualized consideration (Carr, 2011; Dexter, 2018; Dexter & Richardson, 2020). Empirical studies have reported positive associations between principals’ transformational leadership behaviors and technology adoption (Vermeulen et al., 2015, 2016; Yamamoto & Yamaguchi, 2019). Core dimensions often discussed include idealized influence, inspirational motivation, intellectual stimulation, and individualized consideration (Lindqvist, 2019; Puspitasari et al., 2024). Recent evidence from Swiss high school contexts also suggests that, while principals may share student-centered orientations when driving digital transformation, transformational leadership can be more effective than transactional approaches in enabling sustained change (Ruloff & Petko, 2025). The existing literature has proposed a range of leadership functions that are particularly salient for digital transformation. Principals may be expected to act simultaneously as visionary leaders, instructional leaders, and technology facilitators (Dexter & Richardson, 2020; Dagli et al., 2023). In pressure-filled transformation processes, sustaining teachers’ motivation and professional learning—at both individual and collective levels—has been highlighted as crucial (Banoğlu et al., 2023). Correspondingly, principals’ support may include not only professional learning opportunities and instructional guidance but also emotional support that maintains morale and effectiveness amid uncertainty (Tshelane, 2015; Karakose et al., 2021). Recent evidence also points to leader characteristics such as empathy, adaptability, and proactivity as being important for AI-related implementation in schools (Pietsch & Mah, 2025). Alongside these relational and motivational aspects, a prominent strand of research emphasizes principals’ own digital competence and their technology-related pedagogical knowledge as key resources for leading technology integration and advancing school-level innovation (Tshelane, 2015; Dagli et al., 2023). Further, principals’ perceived utility of digital resources, their vision, their own digital literacy, and the external support they receive have been confirmed as key factors influencing the success or failure of transformation (Navaridas-Nalda et al., 2020; Yuliandari et al., 2023; Eickelmann & Drossel, 2020). Additionally, the principal’s age and years of teaching experience, the school’s digital culture, and teachers’ perceptions also influence the school’s digital transformation (Navaridas-Nalda et al., 2020; Al Nuaimi et al., 2024).

AI has the potential to reduce administrative burdens and enhance management efficiency, but it may also erode or even replace certain traditional leadership functions (Holmes et al., 2019). The International Society for Technology in Education (2024) outlines standards for educational administrators as technology leaders, emphasizing equitable access to technology for both teachers and students and the safe, ethical, and lawful use of digital tools. Dagli et al. (2023) noted that school principals express concerns about the high costs of emerging technologies such as AI and the metaverse, as well as apprehensions regarding parents’ views on integrating AI into education. Accordingly, these emerging opportunities and risks underscore the need to examine how principals’ perceptions of school digital transformation in the age of GenAI align with the leadership styles they enact in practice.

### 2.3. Contextualizing Principal Leadership in China

Cultural norms profoundly shape the relational and moral foundations of leadership. Weymes (2004) notes that Western organizations tend to emphasize individual rights and freedoms, whereas many Asian organizations display more family-like characteristics—where an “excellent leader” is likened to an excellent father, responsible for members’ development while also expecting loyalty and responsiveness. In China, conceptions of leadership are deeply shaped by Confucian traditions, in which self-cultivation, moral virtue, and personal character form the core basis of authority and legitimacy (Chen, 2017; Ge et al., 2024). In school, this moral–relational orientation often takes the form of “benevolent parental” leadership, with principals’ authority deriving not only from formal position, but also from visible care and moral standing (Liu et al., 2023; Walker & Qian, 2022). Meanwhile, recent governance agendas emphasize family-school-community collaboration, prompting schools to institutionalize parental participation through formal structures and regular communication. Yet exam-driven accountability amplifies parents’ expectations for test performance, reinforcing a short-term, performance-oriented climate and making parent-related pressures a salient consideration in principals’ reform leadership (Wang et al., 2025).

China’s socio-political and governance landscape adds a further layer of complexity. The state plays a strong steering role in education reform (Huang et al., 2015; Walker & Qian, 2022); yet under a “centralized–decentralized” governance model, local governments and schools retain latitude to interpret and enact policies (Turner, 2017; Brien, 2017). Public school principals are appointed and evaluated by local education authorities and therefore operate under local governmental leadership and administrative oversight (Qian et al., 2017; Hawkins, 2000). Walker et al. (2012) found that Chinese principals were particularly concerned with financial responsibility and resource acquisition, as well as maintaining relationships with government officials. Relatedly, Li et al. (2025) identify “guanxi” as a distinctive dimension of Chinese principal leadership: a network of ties that can be mobilized when needed and through which one may exert influence on behalf of others. The operation of guanxi reflects Chinese principals’ agency in mobilizing and leveraging relational networks to advance school priorities (Leithwood et al., 2020).

Moreover, cross-national analyses indicate persistent digital divides between high-income and developing contexts (Van de Werfhorst et al., 2022). In many developing regions, weak infrastructure and limited access to core technological resources remain key bottlenecks for integrating AI and related technologies into schools (Nhung & Huong, 2025; Purwanto et al., 2020; Aruleba & Jere, 2022). Against this backdrop, China, as the world’s largest developing country, faces a pressing need to strengthen school infrastructure and ensure principals perceive adequate access to digital resources.

In sum, Chinese principals operate in a distinctive and highly complex educational context. Examining how they make sense of GenAI-era digital transformation, construct their roles amid intersecting pressures, and enact leadership in response is crucial for capturing the emerging diversity of principal leadership configurations in the global wave of digital transformation.

## 3. Method

**Figure 1:**
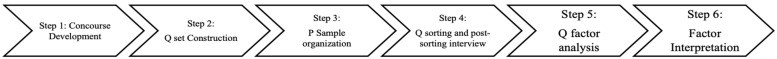
Research process.

This study employed Q to uncover Chinese K–12 principals’ subjective perceptions of school digital transformation in the GenAI age and analyze the leadership styles embodied in those perceptions. Q methodology can capture participants’ subjective orientations and authentic viewpoints, while also generating new hypotheses about human subjectivity (Gil et al., 2020). This study followed the Q methodology steps shown in Figure 1: (1) Concourse development; (2) Q set construction; (3) P sample organization; (4) Q sorting and post-sorting interviews; (5) Q factor analysis; (6) factor interpretation.

### 3.1. Development of the Concourse

The construction of the data collection instrument in Q, a set of items to be ranked by participants, is crucial to the process. The process begins with collecting the concourse, which refers to the comprehensive set of all statements related to a research topic, encompassing everything that can be said about the subject matter and shared within a given culture or society (Watts & Stenner, 2012, pp. 49–51). This study employed a combination of literature review and focus group interviews to collect statements and develop the Q concourse. First, we drew on relevant literature and academic journals focusing on “principal leadership,” “digital transformation,” and “generative artificial intelligence” to extract 98 statements related to the research theme. Second, we recruited six principals to participate in focus group interviews, where the questions centered on their perspectives on the educational applications of generative AI, their perceptions of the roles they play in leading school digital transformation, and their practical experiences in this process. All interviews were conducted online via Tencent Meeting. The participants included three males and three females; three had less than five years of leadership experience, while the other three had five or more years; two were primary school principals, and four were middle school principals. We repeatedly reviewed and analyzed the interview transcripts to ensure that the statements truly reflected the participants’ subjective perceptions. Ultimately, 50 statements were extracted from the interviews and integrated with the 60 preliminarily collected statements, resulting in a total of 158 statements for the Q concourse.

### 3.2. Construction of the Q Sample

The final set of statements generated from the concourse is called the Q sample. Categorizing the Q concourse is an effective method for Q sample selection (Watts & Stenner, 2012, pp. 59–60). To develop the Q sample, we followed a three-step process: categorization, review, and final selection. First, the research team categorized the 158 collected statements in the Q concourse into thematic groups, including (a) vision-setting; (b) principals’ digital competence; (c) teacher support and capacity building; (d) school culture and participation mechanisms; (e) external collaboration and stakeholder management (e.g., parents); (f) policy–resource conditions and structural constraints; and (g) perceived value of GenAI for educational improvement. After categorizing the statements, each researcher independently selected statements, and we included only those jointly chosen by more than two researchers; this process reduced the number of statements to 45. Finally, the research team conducted multiple rounds of iterative review collectively to ensure the relevance, representativeness, and clarity of the items, ultimately forming a Q sample consisting of 30 statements.

### 3.3. P Sample Organization

The P sample refers to the respondents participating in the Q-sorting process. Q does not claim universality for the population but instead emphasizes the purposeful selection of participants who can provide profound insights into the research topic (Watts & Stenner, 2012, pp. 73–76). We recruited principals from a provincial capital city in eastern China, where the local government has explicitly prioritized educational digital transformation and where several leading AI companies are clustered. This setting helped ensure that participating principals had direct exposure to, and practical familiarity with, school digital transformation in the age of GenAI. We extended invitations to 30 primary and secondary school principals in the case region, with 23 principals consenting to participate. To ensure procedural rigor and minimize social desirability bias, the researchers provided standardized instructions and emphasized the voluntary nature of participation, along with anonymity and confidentiality. All participants provided written informed consent. These demographic details are summarized in Table 1.

### 3.4. Q Sorting and Post-Sorting Interviews

**Figure 2:**
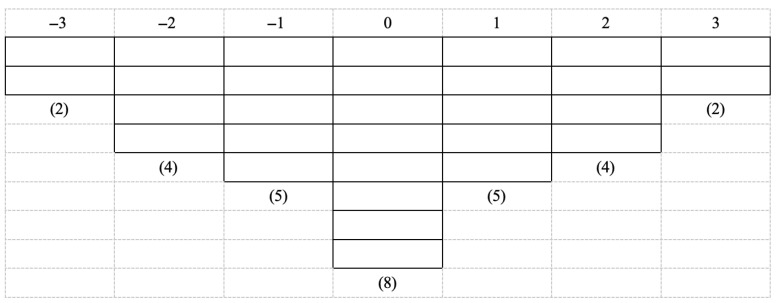
Sample grid for Q sorting pattern.

At the core of Q methodology is the Q sort, in which P-samples are invited to rank a set of topic-specific subjective statements within a forced-distribution grid from “most like me” to “least like me” (Watts & Stenner, 2012, pp. 78–80). A secure, web-based Q-sorting platform was developed for this study to enable participants to complete the Q sort remotely. The platform allowed participants to view all 30 Q statements in full and to drag and place each statement into any position on the forced-distribution grid. The participants were required to read all 30 Q statements and, based on their subjective perceptions, rank them on a scale from −3 (strongly disagree) to +3 (strongly agree), placing the statements into a normal distribution matrix, as illustrated in Figure 2. Q sorting centers on relative prioritization rather than absolute ratings for individual statements. Accordingly, Q studies commonly use a quasi-normal forced-distribution grid, placing more statements near the midpoint and fewer at the extremes (Watts & Stenner, 2012, pp. 16–17). For a 30-statement Q set, a wider scale (e.g., −4 to +4) would increase judgment burden and may induce fatigue and more arbitrary placements, whereas a narrower grid (e.g., −2 to +2) would provide insufficient discrimination, leading many statements to cluster in the middle categories and reducing factor analytic sensitivity to distinct subjective configurations. The 7-point grid (−3 to +3) therefore offers a more appropriate balance between discrimination and stability, helping constrain participants to make meaningful trade-offs and produce more reliable sorting results. All 23 principals completed the online Q-sort individually via the web-based system. Upon finishing the sorting, participants were invited to review and verify their rankings before final submission. Each principal typically required approximately 15–20 min to complete the Q-sort. All 23 principals completed the online Q-sort individually via the web-based system. Upon finishing the sorting, the participants were invited to review and verify their rankings before final submission. Each principal typically required approximately 15–20 min to complete the Q-sort.

Following the Q sort, we conducted follow-up interviews to elicit participants’ reflections and thereby deepen the analysis (Watts & Stenner, 2012, pp. 81–83). This process has been proven useful in providing deeper and more comprehensive qualitative descriptions (Militello et al., 2014). We conducted the interviews via Tencent Meeting after each participant completed the sorting task, with the average interview duration being 1.5 h. The interviews primarily focused on the following questions: (1) Can you describe your management experience and the current state of school digital transformation? (2) Why did you choose the two most important statements? (3) Why did you choose the two least important statements? (4) How did you consider the second-most important and second-least important statements? (5) Could you share how you lead or respond to school digital transformation? (6) What are your views and attitude toward integrating GenAI into education?

### 3.5. Data Analysis

The Q sorts from participants were entered into the PQMethod (version 2.35) software for analysis. The critical α value was set to *p* < 0.05. We employed principal component factor analysis to extract eigenvalues. In accordance with the criteria proposed by Watts and Stenner (2012, pp. 93–105), the factors must meet the following requirements: (1) an eigenvalue greater than 1; (2) a cumulative explained variance of 35–45% or higher; (3) and each factor being represented by at least two participants. Based on the results, the first four factors were selected, with a cumulative variance explanation ratio of 56%, meeting the basic requirements of Q analysis. We calculated the Z-scores and Q-sort values for each type and statement.

### 3.6. Q Factor Interpretation

To further elucidate the distinctions between factors and the perspectives derived from the analysis, we examined statements relative to one another, including the highest and lowest ranked statements, distinguishing statements across the factor interpretation process.

## 4. Results

### 4.1. Analysis Results

**Figure 3:**
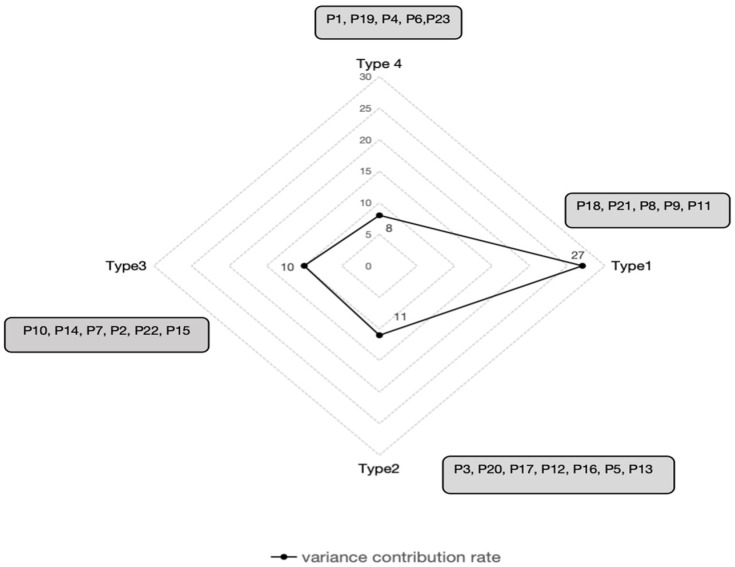
Eigenvalues and explained variances for four factors.

Four distinct types of perceptions regarding digital transformation were identified through factor analysis, revealing four corresponding leadership styles among principals in the school digital transformation context. The variance explained by each type was 27% for Type 1, 11% for Type 2, 10% for Type 3, and 8% for Type 4, resulting in a cumulative explained variance of 56% across the four perception types. Five participants (P18, P21, P8, P9, P11; 21.7% of all participants) were significantly associated with Type 1; seven participants (P3, P20, P17, P12, P16, P5, P13; 30.4% of all participants) loaded significantly on Type 2; six participants (P10, P14, P7, P2, P22, P15; 26.1% of the total) were significantly associated with Type 3; and five participants (P23, P1, P19, P4, P6; 21.7% of the total) were significantly associated with Type 4 (see Figure 3). The four-factor solution demonstrates statistically significant distinctiveness. Table 2 presents the statement-by-statement Z-scores and Q-sort values for each of the four types. The Z-scores represent standardized results from factor analysis, whereas the Q-sorted values indicate the relative ranking of each statement based on participants’ levels of agreement or disagreement during the Q-sorting process. This study focuses on Q-sorted values, especially the statements with the highest and lowest scores, to capture and analyze the characteristics of each group and their corresponding leadership types.

### 4.2. Principals’ Perceptions and Leadership Styles

We identified four principal types based on attitudes and leadership styles toward digital transformation. The following sections elaborate on each type in turn, detailing their demographic profiles, consensus and divergence on key statements, and supporting interview evidence.

#### 4.2.1. Type 1: Cautious Observation–Technological Gatekeeping Leadership

Principals of this type maintain a cautious stance toward GenAI applications in education, emphasizing the value of traditional experience and acting as strategic gatekeepers of educational technology.

This group showed the highest agreement with statements Q29 (“The principal should not depend solely on digital transformation to enhance teaching quality but also focus on the effectiveness of traditional teaching methods”) and Q28 (“The principal should not overly rely on tech to lead school transformation but also value teachers’ opinions and past development experience”). Together, these top-ranked statements characterize a leadership orientation that prioritizes teaching effectiveness and accumulated managerial experience while adopting a cautious approach to technology adoption. For example, P18 said, “We are responsible for our students’ Gaokao performance. New technological tools keep emerging, and as principals we cannot blindly follow trends. School development should be steadily built on the long-term experience we have accumulated.” Principal P8 added, “Most of our students are children who have moved with migrant-worker parents. Their family circumstances are relatively difficult, and parents are mainly concerned about academic performance. If we overemphasize technology use at school, parents may not accept it.”

In contrast, Type 1 strongly rejected culture- and expansion-oriented approaches, including Q6 (“The principal should foster an open, innovative school culture to enhance teachers’ confidence in using smart technologies”) and Q11 (“The principal should actively collaborate with education experts, tech firms, and other schools to explore new digital transformation models and innovation methods”). This pattern suggests that, relative to other priorities, building an explicitly innovation-oriented culture and pursuing extensive external collaboration were not emphasized in this factor. P11 explained: “There is too much uncertainty surrounding GenAI. I think we should wait and see for a while, and only incorporate it into the school’s teaching and management once the technology becomes more mature.” Moreover, principals in this type also assigned low scores to vision- and exemplar-focused leadership statements, such as Q2 (“The principal’s core role is to shape the school’s digital transformation vision and cultivate team trust”) and Q3 (“The principal’s beliefs and positive attitude are crucial for driving the school’s digital transformation”). As P9 put it, “Although the government provides policy and financial support, advancing digital transformation still requires us to proactively compete for limited external resources. We also need to consider whether GenAI can genuinely improve students’ academic performance […] I have not made digital transformation the core task of our school’s reform.”

#### 4.2.2. Type 2: Moderate Ambition–Culturally Transformative Leadership

Principals of this type advance digital transformation by reshaping school culture, cultivating an open and supportive organizational environment, and building shared commitment among teachers toward GenAI-enabled change.

This group of principals ranked Q6 (“The principal should foster an open, innovative school culture to enhance teachers’ confidence in using smart technologies”) the highest. For example, P17 drew on her school’s motto to explain: “Our motto is ‘Harbor ambitions for the world; be masters of the future’, which is meant to guide us in embracing the changes of the times and transformations in education. […] Our educational philosophy, ‘Teach comprehensively, cultivate talent broadly’, emphasizes broadly accepting changes in society.” She further added, “Digital transformation aligns very well with our campus culture.” Principal P12 also underscored the importance of culture. He said, “What teachers are really afraid of is not the technology itself, but whether something will go wrong if they don’t use it well—and whether they will be judged for it. I would first make the bottom lines and guiding principles clear. As long as it does not violate our educational goals, teachers can try boldly […] GenAI is not something we bring in today and roll out across the whole school tomorrow. I’d rather move more slowly, so teachers can shift from being willing to try, to using it smoothly, and then to using it with confidence.” Consistent with this culture-centered approach, Type 2 also strongly endorsed the principal’s role in vision-building and trust cultivation, with Q2 (“The principal’s core role is to shape the school’s digital transformation vision and cultivate team trust”) receiving a high score.

In contrast, Type 2 rejected resource- and stakeholder-balancing framings as central constraints. They most strongly disagreed with Q23 (“Digital transformation success depends on the principal obtaining sufficient tech support and financial resources”) and Q20 (“The principal needs to balance parents’ different expectations of digital education”). Across the interviews, principals commonly noted that policy direction from the government has become increasingly clear and that many GenAI tools are now easier to access. They emphasized that the key challenge lies in using these tools appropriately so that they serve teaching rather than create disruption. P16 stated explicitly, “Of course we should listen to parents’ views, but I don’t see this as a matter of ‘balancing different expectations.’ What matters more is that the school makes the direction clear: times are changing, and education has to keep up […] It is also important to secure support from some influential parents. They can provide material support in terms of equipment, platform resources, and opportunities for extracurricular practice; more importantly, their understanding and endorsement can create a demonstration effect among the wider parent community and offer a form of ‘legitimacy backing’ for the school’s reform.” Across interviews, Type 2 principals also noted that maintaining constructive ties with local education authorities was important for securing policy alignment and procedural support for GenAI-related initiatives.

#### 4.2.3. Type 3: Moderate Ambition–Emotionally Empowering Leadership

Principals of this type prioritize emotional support and relationship maintenance, using care-based interactions and professional development to sustain teachers’ engagement with GenAI-related digital transformation.

Q5 (“In the school’s digital transformation, the principal should boost teachers through emotional support and care to overcome challenges”) was rated highly. P7 explained, “I feel that rather than enforcing something, what’s more important is a deep connection between people. If teachers emotionally accept you and accept the school’s digital transformation policies, then the principal doesn’t need to invest extra time and energy.” She further added, “We need to be wary of digitizing people and behaviors in the process of digital transformation—I find that very scary. […] When I talk with teachers, I usually don’t start by saying, ‘You need to build your capacity’ or ‘You need to be self-driven.’ I’d rather begin by asking, ‘Have you been exhausted lately? What concerns do you have? What can I do to help?’”. Type 3 also placed strong emphasis on principals’ personal beliefs, with Q3 (“The principal’s beliefs and positive attitude are crucial for driving the school’s digital transformation”) receiving the highest scores. For example, P10 noted, “I may not be able to keep up with younger teachers in learning the technology, but my attitude matters. It’s my job to give teachers confidence.”

Type 3 showed the lowest agreement with Q23 (“Digital transformation success depends on the principal obtaining sufficient tech support and financial resources”) and Q25 (“As a principal, I believe I have the ability to deal with challenges in the school’s transformation process”). For example, P14 stated, “The costs of integrating GenAI into teaching and school management are not high, and government support is already in place. […] So technology and funding are not the key constraints; principals need to be proactive and mobilize support from multiple sources.” He further emphasized, “GenAI-powered education is an inevitable trend, but it needs to be integrated gradually. For example, I myself also work as a Chinese language teacher—while I’m full of enthusiasm for technology, I lack professional technical knowledge. My strength is that I know what difficulties teachers are facing and what help they need […] I see myself as leading a big family. I encourage teachers to try first, and if problems arise, I will take responsibility and provide a safety net, so no one has to bear the risk alone.”

#### 4.2.4. Type 4: High Aspiration–Strategy-Driven Leadership

Principals of this type display high aspirations for GenAI’s potential and drive AI-enabled digital transformation through strategic planning, clear goal-setting, and values-based mobilization that signals strong leader commitment.

This group gave the highest scores to Q1 (“The principal should exemplify through personal conduct and values, inspiring teachers to jointly participate in digital transformation”) and Q4 (“Clear digital transformation goals and vision are essential to motivate teachers to actively embrace technological change”). Interview accounts reinforced this strategic orientation. P1 stated, “GenAI applications in education represent a rare opportunity to enhance school quality. […] I have already incorporated digital transformation into our school’s five-year development plan.” He added, “I specifically organized a two-month series of ‘collective vision workshops’ and arranged for teacher representatives to visit AI-education benchmark schools for observation and learning. Ultimately, building on our existing cultural identity of ‘Scholarly spirit for enduring growth’ (书香致远), we jointly distilled a transformation vision: ‘Intelligence-empowered scholarly culture, wisdom-guided nurturing of new generations’ (智启书香,慧育新人). […] This process both anchored the school’s AI direction and preserved its cultural identity, and was repeatedly referenced as facilitating teachers’ identification with the reform agenda.” Q3 (“The principal’s beliefs and positive attitude are crucial for driving the school’s digital transformation”) and Q11 (“The principal should actively collaborate with education experts, tech firms, and other schools to explore new digital transformation models and innovation methods”) were also relatively well endorsed by this type. For example, P23 mentioned, “The resources the government can provide are ultimately limited. […] Our goal is to build a model school for digital transformation, and a large part of my work involves negotiating partnerships with key leaders in technology companies to jointly explore pathways for integrating GenAI into teaching and school management.”

The statements this group disagreed with most were Q29 (“The principal should not depend solely on digital transformation to enhance teaching quality but also focus on the effectiveness of traditional teaching methods”) and Q7 (“The principal should be a hands-on practitioner of smart technology applications, setting an example in digital transformation”). P19 explained, “What teachers really need is a signal: whether the school is genuinely committed to doing this, and whether the leadership is trustworthy and dependable. When I make it clear why we are doing it, how far we will go, and what we will not do, teachers are much more willing to follow and engage.” P6 added, “Teachers have a great deal of trust in me. I don’t need to be a technical expert; rather, I need to tell teachers clearly that GenAI applications in education relate to the future development of our students—and even of humanity—and therefore we must embrace them proactively.”They also did not agree with Q5 (“In the school’s digital transformation, the principal should boost teachers through emotional support and care to overcome challenges”). P4’s comments aligned with this pattern: “In schools, people care more about who you are—whether you are fair, whether you take responsibility, and whether you put students first. As long as they trust your values and intentions, reform tends to move forward more smoothly. Compared with that, I don’t think emotional support is the most crucial element.”

## 5. Discussion and Conclusions

Using Q methodology, this study examines Chinese K–12 principals’ perspectives on school digital transformation in the age of GenAI and explores the leadership styles associated with these understandings. Consistent with prior scholarship, our findings further underscore the fact that principal leadership is a central determinant of both the trajectory and effectiveness of school digital transformation (Carr, 2011; Navaridas-Nalda et al., 2020; Yuliandari et al., 2023; Ghavifekr & Pei, 2023). In a context where GenAI is accelerating shifts in the educational ecosystem, Chinese principals vary in how they construct leadership roles and interpret the meaning of their practices. In this study, we identified four different perception–leadership styles: Cautious Observation–Technological Gatekeeping Leadership, Moderate Ambition–Culturally Transformative Leadership, Moderate Ambition–Emotionally Empowering Leadership, and High Aspiration–Strategy-Driven Leadership. The findings suggest that, while these four leadership styles resonate with core elements highlighted in Western models of effective leadership, such as transformational leadership and digital leadership, they also embody distinctive features that are deeply rooted in China’s educational context (Walker & Qian, 2018; Qian et al., 2017).

Principals characterized by Cautious Observation–Technological Gatekeeping Leadership (Type 1) positioned themselves as “gatekeepers” who regulate and safeguard the use of technology in their schools. Driven by concerns about students’ cognitive development and academic performance, these principals argued that introducing AI should be preceded by rigorous value-based screening and careful assessments of contextual fit. This concern aligns closely with prior evidence that heightened attention to standardized test performance functions as a constraining, high-priority consideration for Chinese principals when advancing educational reforms (Li et al., 2025; Wang et al., 2025). They adopted a relatively conservative stance toward the new wave of digital transformation prompted by GenAI, emphasizing the irreplaceability of established pedagogical approaches and accumulated managerial experience. This tendency echoes reflective and critical scholarly discussions of GenAI in education (Selwyn, 2022; Zhao et al., 2024).

In sharp contrast to Type 1, High Aspiration–Strategy-Driven Leadership (Type 4) reflects a markedly more forward-looking orientation. These principals displayed a proactive embrace of school digital transformation in the age of GenAI, emphasizing the need to seize strategic opportunities amid the accelerating digitalization of education. They typically articulated clear, school-level strategic plans for digital transformation. At first glance, this leadership style appears to align with Western models of transformational leadership (Lindqvist, 2019). However, a closer examination reveals a distinctive configuration of leadership practice. In shaping their vision, they adapted official guidelines to align with the school’s collectively shared value commitments, thereby securing teachers’ identification with and buy-in to the transformation agenda. This emphasis on mission setting as a symbolic and moral compass resonates with Confucian traditions and is consistent with Ng et al.’s (2015) argument that leadership vision in many Asian contexts is often simultaneously moral in orientation and grounded in collectivist commitments. In addition, unlike prior studies that emphasize principals’digital competence and technological pedagogical content knowledge as key resources for leading school digital transformation (Dagli et al., 2023; Tshelane, 2015), these principals’ modelling influence was expressed primarily through their stance rather than through hands-on technology use itself. More specifically, they viewed the leader’s core role as signaling a positive stance toward GenAI use in education and mobilizing teachers’ active engagement through values-based identification. This pattern also corroborates prior research suggesting that, under Confucian cultural influence, Chinese principals’ authority tends to rest more on moral cultivation and personal character than solely on professional knowledge and technical competence (Walker & Qian, 2022).

Principals characterized by Moderate Ambition–Culturally Transformative Leadership (Type 2) and Moderate Ambition–Emotionally Empowering Leadership (Type 3) styles both expressed a stance of skeptical optimism toward school digital transformation in the age of GenAI. They acknowledged AI’s potential to enhance instructional and administrative efficiency, yet did not incorporate GenAI adoption into their schools’ strategic development plans. Consistent with Kozloski’s (2006) perspective on digital transformation strategy, both groups advocated a step-by-step, incremental approach to implementation in order to mitigate the uncertainty and risks associated with adopting new technologies. Both groups placed strong emphasis on activating teachers’motivation and agency during the transformation process (Banoğlu et al., 2023; Karakose et al., 2021), yet they differed markedly in how this was enacted in practice. Type 2 principals foregrounded school culture reconfiguration: by cultivating an organizational culture that is open and inclusive, encourages innovation, and tolerates trial and error, they sought to strengthen teachers’ psychological acceptance of GenAI for teaching and their adaptive capacity for change. However, unlike Western leadership models that prioritize individual teacher autonomy and peer collaboration, these principals placed greater emphasis on building reform consensus by reinforcing teachers’ moral responsibility and collective sense of belonging (Li et al., 2025). In contrast to Type 2’s culture-centered approach, Type 3 principals prioritized relationship maintenance, placing the harmony and stability of teacher–student and home–school relationships at the core of their leadership practice in the digital age. In contrast to the communication approaches commonly emphasized in Western contexts, which foreground teacher professional development and empowerment (Barnett & McCormick, 2004; Moye et al., 2005), Type 3 principals were more inclined to adopt an interactional mode characterized by paternalistic care. This emotion-centered leadership orientation reflects the cultural ethos of qing and relational obligation that characterizes many Chinese educational organizations, and it aligns with scholarship highlighting the centrality of “benevolent authority” in school leadership in the Chinese context (Chen, 2017; Liu et al., 2023).

Furthermore, in contrast to research on educational digital transformation in many developing-country contexts that identifies inadequate infrastructure and limited technological access as core constraints (Purwanto et al., 2020; Aruleba & Jere, 2022; Nhung & Huong, 2025), the principals in this study generally reported that earmarked central government funding and local institutional support had largely alleviated concerns about hardware provision. As a result, infrastructure was not treated as a primary consideration in their leadership decision-making. However, principals of Types 2, 3, and 4 explicitly noted that a key component of their leadership practice involved building and maintaining “guanxi” with local government officials, technology-firm managers, and influential parent representatives in order to secure support and facilitate the smooth advancement of school digital transformation. It should be emphasized that guanxi in the Chinese context differs fundamentally from the interpersonal networking typically discussed in Western scholarship on school leadership (Leithwood et al., 2020). As Li et al. (2025) note, in centralized governance systems, educators’ relational networks are particularly consequential. Principals often leverage guanxi to cultivate external ties that secure resources and institutional legitimacy. These networks are not merely channels for administrative coordination; they also enable resource mobilization, risk management, and strategic and legitimacy support, thereby illustrating principals’ agentic capacity to advance reform (Leithwood et al., 2020).

## 6. Implications

Our study advances leadership theory and provides empirically grounded insights into how Chinese K–12 principals navigate GenAI-enabled change amid emerging opportunities and risks. The findings of this study offer several implications for both theory and practice.

First, GenAI’s black-box features and accompanying ethical controversies contribute to a complex and differentiated pattern of principals’ attitudes toward school digital transformation in the GenAI era. Overall, Chinese K–12 principals’ attitudes toward GenAI in education are not simply supportive or opposed; instead, they form a continuum from conservative and cautious to skeptically optimistic and, ultimately, highly expectant. Accordingly, when measuring or interpreting principals’ attitudes toward GenAI, researchers should avoid relying solely on a single dimension. Instead, greater attention should be paid to the internal structure of principals’ attitudes, including configuration differences, trade-off logics, and priority ordering. Such an approach can more accurately capture principals’ genuine stances and governance orientations under the uncertainty associated with GenAI.

Second, this study further underscores the need to examine principal leadership theory within non-Western contexts. Specifically, principals’ role conceptions are jointly shaped by Confucian traditions that emphasize relational ethics and moral order; a governance framework combining centralized steering with local discretion in education administration; institutional arrangements through which local officials appoint and supervise principals; and performance-based accountability regimes centered on students’ academic achievement. These contextual forces, in turn, give rise to several distinctive enactments of leadership, including prioritizing students’ academic performance and cognitive development in technology adoption decisions (Type 1), framing vision in moral and collectivist terms (Types 4 and 2), exercising influence primarily through values-based signaling and personal character rather than hands-on technical modeling (Type 4), and stabilizing change through affective support characterized by “paternalistic care” (Type 3).

Third, this study highlights the strategic value of building guanxi-based networks in the context of digital transformation. Unlike in many Western settings where principals’ professional networks primarily facilitate administrative communication and collaboration, guanxi networks in China often simultaneously enable resource mobilization, risk mitigation, and legitimacy support. As such, guanxi capital constitutes an indispensable analytical lens for understanding principal leadership in China. Particularly in the digital age, principals need to leverage guanxi to secure scarce government resources and to connect with technology firms and influential parent groups in order to advance reform. Yet guanxi, as a pivotal mechanism for mobilizing resources, is frequently overlooked or downplayed in mainstream Western theories of educational leadership (Li et al., 2025).

Practically, the findings of this study indicate that, supported by dedicated government investment and institutional safeguards, traditional “hard” constraints such as insufficient infrastructure have been significantly alleviated. By identifying distinct perception–leadership types, this study offers a technology-oriented account of leadership strategies and shows how GenAI-related perceptions translate into differentiated approaches to leading transformation. Accordingly, more targeted support strategies should be developed to align with the distinctive profiles of different principal types. With Technological Gatekeeping principals, the priority is transparent and participatory boundary-setting, reinforced by clear accountability and verification routines so that risk control does not constrain teacher autonomy or innovation. Where principals are Culturally Transformative, support should institutionalize shared norms and strengthen psychological safety, enabling genuine experimentation rather than symbolic compliance. For Emotionally Empowering principals, what matters most is relational leadership under uncertainty, alongside timely support that sustains teachers’ confidence and self-efficacy in everyday AI use. By contrast, Strategy-Driven principals call for a dual-track approach that couples innovation support with robust safeguards for accountability and fairness, protecting teachers’ rights and discretion so that rapid diffusion of generative AI does not undermine procedural justice or professional autonomy.

## 7. Limitations and Future Perspectives

This study has several limitations that suggest directions for future research. First, it examines leadership for GenAI-era school digital transformation only from principals’ perspectives and relies on self-reports, which may be subject to social desirability bias. Future work could incorporate multiple stakeholders, such as teachers and students, and include more on-site observations. Triangulating evidence across multiple data sources would enable a more comprehensive and in-depth understanding of principal leadership styles. Second, all participating principals were drawn from a region where the local government strongly prioritizes educational digital transformation and where leading AI companies are concentrated. While this sampling feature strengthened the study’s contextual depth, it may limit the transferability of the findings to resource-constrained schools, rural areas, and other settings with markedly different socioeconomic and cultural conditions. Accordingly, nationwide cross-regional comparative studies could examine how principals’ understandings and leadership practices for school digital transformation vary across regions with different levels of economic development, offering both theoretical and practical insights. Third, a cross-sectional design was adopted in this study and therefore only captures principals’ perceptions and leadership style characteristics at a single point in time. Given the rapid pace of AI development and educational digital transformation, future research should adopt longitudinal designs to examine how principals’ understandings of AI-driven change evolve and how their leadership styles adapt over time.

## Figures and Tables

**Table 1:** Demographic background.

No.	Gender	Age	School Level	Management Experience
P1	Female	56	Senior high school	16
P2	Male	54	Senior high school	5
P3	Male	40	Senior high school	1
P4	Male	50	Senior high school	2
P5	Male	40	Junior high school	4
P6	Male	50	Primary school	25
P7	Female	47	Primary school	7
P8	Female	53	Primary school	16
P9	Female	45	Primary school	5
P10	Male	48	Junior high school	5
P11	Male	50	Primary school	17
P12	Male	44	Primary school	6
P13	Male	42	Senior high school	7
P14	Male	40	Junior and senior high school	15
P15	Female	40	Primary school	2
P16	Male	50	Primary school	9
P17	Female	55	Senior high school	11
P18	Male	50	Senior high school	4
P19	Male	40	Primary school	10
P20	Male	57	Junior and senior high school	4
P21	Male	50	Junior and senior high school	12
P22	Male	55	Primary school	15
P23	Male	50	Junior and senior high school	14

**Table 2:** Z-scores and Q-sort values of the statements for each type.

No.	Statement	Type 1		Type 2		Type 3		Type 4	
		Z-Score	Q-Sort Value	Z-Score	Q-Sort Value	Z-Score	Q-Sort Value	Z-Score	Q-Sort Value
Q1	The principal should exemplify through personal conduct and values, inspiring teachers to jointly participate in digital transformation.	−0.718	−1 *	0.907	2	1.263	2	2.253	3 *
Q2	The principal’s core role is to shape the school’s digital transformation vision and cultivate team trust.	−1.527	−2 *	2.23	3 *	−0.404	0 *	0.847	2 *
Q3	The principal’s beliefs and positive attitude are crucial for driving the school’s digital transformation.	−1.178	−2 *	0.322	0 *	2.216	3 *	1.104	2 *
Q4	Clear digital transformation goals and vision are essential to motivate teachers to actively embrace technological change.	0.121	0	1.077	2	0.588	1	1.632	3
Q5	In the school’s digital transformation, the principal should boost teachers through emotional support and care to overcome challenges.	−0.596	−1	0.071	0	1.563	3 *	−0.86	−2 *
Q6	The principal should foster an open, innovative school culture to enhance teachers’ confidence in using AI.	−1.622	−3	2.238	3 *	1.287	2 *	0.411	0 *
Q7	The principal should be a hands-on practitioner of smart technology applications, setting an example in digital transformation.	−0.412	0	−0.664	−1	0.473	1 *	−1.346	−3
Q8	The principal should organize tech training and seminars to increase teachers’ acceptance of new technologies.	−0.588	−1 *	0.269	0	1.69	−1	0.67	1
Q9	Establishing open communication channels is vital for involving teachers and students in digital transformation.	−0.614	−1	−0.69	−1	1.086	2 *	−0.385	−1
Q10	The principal’s open-mindedness and acceptance of new technologies are crucial for the school’s digital transformation.	0.477	1	0.513	1	0.966	1	0.464	1
Q11	The principal should actively collaborate with education experts, tech firms, and other schools to explore new digital transformation models and innovation methods.	−1.625	−3 *	0.643	2	−0.344	0 *	1.224	2
Q12	The principal should provide customized tech training and support based on teachers’ individual needs to promote tech innovation.	−1.242	−2	0.252	0	−0.32	0	−1.127	−1
Q13	The principal should ensure teachers at all levels get appropriate support and participate in digital transformation.	0.303	1	−0.482	−1	−0.538	−1	−0.181	0
Q14	Digital transformation can significantly enhance the school’s overall effectiveness, and the principal should ensure effective tech and resource integration.	0.279	0	0.106	0	−1.133	−2 *	0.458	1
Q15	GenAI’s educational applications offer unprecedented opportunities to enhance the school’s educational quality and competitiveness.	−0.024	0	0.006	0	−0.663	−1	0.501	1
Q16	The complexity of tech applications and differences in teachers’ digital literacy are key challenges in digital transformation, and the principal needs to offer extra support.	−1.005	−2	−1.327	−2	−0.27	0	−0.222	0
Q17	Smart technology applications can improve educational quality, but schools must address related resource and equipment issues.	0.486	1	0.495	1	−0.38	0 *	0.843	2
Q18	Policy support and government resource guarantees are crucial for digital transformation, and the principal should actively seek policy support.	0.092	0	0.43	1	−0.437	−1	0.49	1
Q19	Teachers’ and parents’ recognition and support are crucial for the principal to exert leadership.	0.039	0	−0.747	−1	−0.023	1	−1.259	−2
Q20	The principal needs to balance parents’ different expectations of digital education.	−0.579	−1	−1.693	−3	−1.194	−2	−2.177	−2
Q21	Policy requirements and resource constraints may limit the principal’s leadership in digital transformation.	0.292	0	−1.289	−2 *	−0.158	0	0.16	0
Q22	The principal should keep learning and improving to lead the school’s digital transformation.	0.659	2	−0.225	−1	−0.674	−1	0.444	0
Q23	Digital transformation success depends on the principal obtaining sufficient tech support and financial resources.	0.112	0	−1.534	−3	−1.897	−3	0.177	0
Q24	The principal needs to keep improving digital literacy to meet the demands of digital transformation.	0.787	2	0.253	0	−0.716	−2	−0.953	−1
Q25	As a principal, I believe I have the ability to deal with challenges in the school’s transformation process.	0.434	1	−1.387	−2	−1.4	−3	0.163	0
Q26	The principal should regularly communicate with teachers, assess digital transformation progress, and adjust strategies to ensure goals are met.	0.625	1	0.578	1	−0.525	2	−0.435	−1
Q27	The principal should have a positive attitude toward smart technology applications, seeing them as opportunities to improve educational quality.	1.122	2	0.649	2	−0.849	−2 *	0.075	0
Q28	The principal should not overly rely on tech to lead school transformation but also value teachers’ opinions and past development experience.	1.957	3 *	−1.468	−2 *	−0.19	0	−0.227	−1
Q29	The principal should not depend solely on digital transformation to enhance teaching quality but also focus on the effectiveness of traditional teaching methods.	2.186	3 *	0.602	1	1.022	1	−1.411	−3 *
Q30	When promoting digital transformation, the principal should create an environment that encourages criticism, giving teachers space for independent exploration and innovation.	1.759	2 *	−0.132	0	−0.039	0	−1.335	−2 *

* *p* < 0.05.

## Data Availability

The data presented in this study are available upon request from the corresponding author. The data are not publicly available due to confidentiality and ethics reasons.
